# Thrombosis among 1537 patients with *JAK2^V617F^*‐mutated myeloproliferative neoplasms: Risk factors and development of a predictive model

**DOI:** 10.1002/cam4.2886

**Published:** 2020-01-28

**Authors:** Yuhui Zhang, Yuan Zhou, Yingshao Wang, Guangshuai Teng, Dapeng Li, Yan Wang, Chenxiao Du, Yafang Chen, Huiqin Zhang, Yanqi Li, Lixia Fu, Kangyin Chen, Jie Bai

**Affiliations:** ^1^ Department of Hematology The Second Hospital of Tianjin Medical University Tianjin China; ^2^ State Key Laboratory of Experimental Hematology Institute of Hematology & Blood Diseases Hospital National Clinical Research Center for Blood Diseases Chinese Academy of Medical Sciences & Peking Union Medical College Tianjin China; ^3^ Tianjin Key Laboratory of Ionic‐Molecular Function of Cardiovascular Disease Department of Cardiology Tianjin Institute of Cardiology Second Hospital of Tianjin Medical University Tianjin China

**Keywords:** *JAK2^V617F^*, myeloproliferative neoplasms, thrombosis

## Abstract

To explore the risk factors of thrombosis in patients with *JAK2^V617F^‐*mutated myeloproliferative neoplasms (MPNs), a cohort of 1537 Chinese patients with *JAK2^V617F^*‐mutated MPN was retrospectively analyzed. The Kaplan‐Meier method and multivariate Cox analysis were used to study the risk factors of thrombosis in patients with *JAK2^V617F^*‐mutated MPN. Among the 1537 MPN patients, 931, 468, and 138 had polycythemia vera (PV), essential thrombocythemia (ET), and primary myelofibrosis (PMF), respectively. The median follow‐up time was 7 years (range 1‐47), and 12.8% of patients (197/1537) died during this period. A total of 16.8% (259/1399) of PV and ET patients had secondary myelofibrosis, and 2.5% (38/1537) of patients developed acute myeloid leukemia (AML). Thrombotic events occurred in 43.9% (675/1537) of patients, among which 91.4% (617/675) were arterial thrombosis and 16.6% (112/675) were venous thrombosis. The number of thrombotic events in PV, ET, and PMF patients was 439 (47.2%), 197 (42.1%) and 39 (28.2%), respectively. The multivariate analysis indicated that age ≥60 years old, HCT ≥48%, at least one cardiovascular risk factor, a history of thrombosis, and *JAK2^V617F^* allele burden (*V617F*%) ≥50% are risk factors for thrombosis in *JAK2^V617F^*‐mutated MPN. According to the results of the multivariate analysis, a risk model of thrombosis was established and comprised low‐risk (0 points), intermediate‐risk (1 points) and high‐risk (≥2 points) groups, among which the incidence of thrombosis was 9.1%, 33.7% and 72.9%. For elderly patients with *JAK2^V617F^*‐mutated MPN and a history of thrombosis, reducing the *V617F%*, controlling HCT and preventing cardiovascular risk factors are necessary measures to prevent thrombosis.

## Graphical abstract

We analyzed the clinical characteristics, laboratory characteristics, cytogenetics, thromboembolism status, disease progression, and overall survival of 1537 Chinese MPN patients with the JAK2*V617F* mutation. The multivariate analysis indicated that age ≥60 years old, HCT ≥48%, at least one cardiovascular risk factor, history of thrombosis, and JAK2*V617F* allele burden (*V617F*%) ≥50% are risk factors for thrombosis of JAK2*V617F*‐mutated MPN. Our study suggested that for elderly patients with JAK2*V617F*‐mutated MPN and a history of thrombosis, reducing *V617F*%, controlling HCT, and mitigating cardiovascular risk factors are necessary measures to prevent thrombosis.

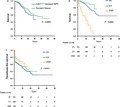

## 1 INTRODUCTION

Philadelphia chromosome‐negative myeloproliferative neoplasms (MPNs) are chronic myeloproliferative diseases caused by clonal expansion of malignant hematopoietic stem/progenitor cells. MPNs mainly include polycythemia vera (PV), essential thrombocythemia (ET), and primary myelofibrosis (PMF).^1^ Thrombosis, leukemic transformation, and myelofibrotic transformation are critical complications of MPN and are highly common in patients with MPN.^2^, ^3^, ^4^, ^5^, ^6^ Fatalities due to thrombosis account for 35%~70% of the total mortality rate of MPN, making it the most important risk factor affecting event‐free survival and overall survival (OS) of MPN patients.^7^

Studies in the last decade have shown that *JAK2*/*CALR*/*MPL* gene mutations are the driver mutations in MPN.^8^, ^9^, ^10^ Among those, the *JAK2^V617F^* mutation is considered the most common driver mutation of MPN and is detected in 95% of PV patients and 60% of ET and PMF patients.^11^

In this work, we analyzed the clinical features, disease progression, and survival status of 1573 MPN patients with *JAK2^V617F^* mutation and explored the risk factors affecting thrombosis in patients with *JAK2^V617F^*‐mutated MPN.

## 2 METHODS

### 2.1 Patients

A cohort of 1537 patients with *JAK2^V617F^*‐mutated MPN from January 1981 to December 2018 at the Second Hospital of Tianjin Medical University and the Institute of Hematology and Blood Diseases Hospital, Chinese Academy of Medical Sciences were selected for analysis. When patients were included, we rechecked their medical history and carefully reevaluated whether they met 2016 WHO diagnostic criteria. All the MPN patients included in this study were diagnosed according to the 2016 WHO diagnostic criteria.^1^ All patients signed an informed consent form. This study was approved by the ethics committee of the Second Hospital of Tianjin Medical University and the Institute of Hematology and Blood Diseases Hospital, Chinese Academy of Medical Sciences.

### 2.2 Analysis of the JAK2^V617F^ mutation and JAK2^V617F^ allele burden

The *JAK2^V617F^* mutation was detected by nested allele‐specific polymerase chain reaction (PCR). The primers are as follows: P_2_5'‐CCTCAGAACGTTGATGGCA‐3', P_2r_5'‐ATTGCTTTCCTTTTTCACAACA‐3', P_nf_5'‐AGCATTTGGTTTTAAATTATGGAGTATATG‐3', and P_mr_5'‐GTTTACTTACTCTCGTCTCCACAAAA‐3'. The PCR amplification conditions were as follows: 95°C for 5 minutes; 40 cycles of 95°C for 30 seconds, 54°C for 25 seconds, and 72°C for 30 seconds; and 72°C for 10 minutes. The PCR products were analyzed by 2.5% agarose gel electrophoresis under ultraviolet light. The products were 453 bp, 279 bp (*JAK2^V617F^* mutant allele), and 229 bp (wild‐type allele). The *JAK2^V617F^*‐positive samples determined by the above methods were detected by StepOne real‐time quantitative PCR with the following primers: forward primer 5'‐AAGCTTTCTCACAAGCATTTGGTTT‐3', reverse primer 5'‐AGAAAGGCATTAGAAAGCCTGTAGTT‐3'; *JAK2^V617F^* wild‐type TaqMan probe VIC‐5'‐TCTCCACAGACACATAC‐3'; and *JAK2^V617F^* mutant TaqMan probe FAM‐5'‐TCCACAGAAACATAC‐3'. The PCR conditions were the same as those listed above. The standard curve was drawn by mixing the *JAK2^V617F^* mutant homozygote with MPN expressing wild‐type JAK2 in different proportions, and the *JAK2^V617F^* allele burden was calculated as follows: *V617F*% = *JAK2^V617F^* mutant/ (*JAK2^V617F^* mutant plus *JAK2^V617F^* wild type).

### 2.3 Group

According to the 2016 WHO diagnostic criteria, the patients were divided into PV, ET and PMF groups. Patients with *JAK^2V617F^*‐mutated MPN were also divided into two groups as follows: V617F% ≥50% and <50%. They were divided according to thromboembolic events into an arterial thrombosis group and a venous thrombosis group. Thromboembolic events in all the patients were verified by computerized tomography scan, magnetic resonance imaging, digital subtraction angiography, or color Doppler ultrasound, and further diagnosed by a specialist. Arterial thrombosis is mainly divided into acute coronary syndrome, cerebral vascular thrombosis, splenic thrombosis, peripheral artery, and other arterial thrombosis, whereas venous thrombosis is mainly divided into peripheral venous thrombosis, portal venous thrombosis, pulmonary venous thrombosis, cerebral venous sinus thrombosis, other venous thrombosis, etc Cardiovascular (CV) risk factors include hypertension, hyperlipidemia, diabetes, and smoking. The secondary myelofibrosis in patients with PV and ET were diagnosed according to the 2016 WHO diagnostic criteria. All the patients in this study underwent bone marrow biopsy and were diagnosed by 2 or 3 hematopathologists independently. Patients with PV, ET, and PMF were divided into risk groups by their respective prognostic scoring systems. ET uses IPSET(International Prognostic Score for ET),^12^ and PMF uses DIPSS‐Plus (Dynamic International Prognostic Scoring System).^13^

### 2.4 Statistical analysis

The overall survival (OS) of MPN patients was defined as the time between the diagnosis of MPN and death or the last follow‐up. Thrombosis‐free, myelofibrosis (MF)‐free and acute myeloid leukemia (AML)‐free survival were defined as the time between the diagnosis and the occurrence of thrombosis, MF transformation and AML, respectively. By comparing the expected survival of a healthy Chinese population with age‐sex matching, using the mortality rate of healthy Chinese as a reference, we calculated the standardized mortality ratio (SMR). The abnormal distribution data are described as the median (range). The qualitative data were compared by the chi‐square test or Fisher's test, and continuous variables were compared with the Mann‐Whitney U test or Kruskal‐Wallis test. The Kaplan‐Meier method was used for survival analysis. Multivariate analysis was performed using the Cox regression model to analyze the risk factors of thrombosis. All statistical data were analyzed using SPSS 24.0 software, and *P* < .05 was considered significant.

## 3 RESULTS

### 3.1 Clinical features of patients with *JAK2^V617F^*‐mutated MPN

The median age at first diagnosis of the 1537 patients with *JAK2^V617F^*‐mutated MPN was 54 (18‐89) years old, 739 (48.1%) were male, and 798 (51.9%) were female. The number of patients with PV, ET, and PMF was 931 (60.6%), 468 (30.4%) and 138 (9.0%), respectively. The proportion of male patients with PV and PMF was higher than that of male patients with ET (*P* < .05). The number of PMF patients with *V617F*% ≥50% and splenomegaly was significantly higher than that of ET and PV patients (*P* < .05). Antiplatelet therapy, such as aspirin, was used in 59.5% of patients (Table 1).

**Table 1:** Clinical characteristics of patients with *JAK2^V617F^*‐mutated MPN

	Total(n = 1537)	PV(n = 931)	ET(n = 468)	PMF(n = 138)	*P*	PV vs ET	PV vs PMF	ET vs PMF
Age media (range)	54 (18‐89)	54 (18‐86)	53 (18‐89)	57 (31‐80)	<0.0001***	0.867	0.002**	0.009**
Age ≥ 60, n(%)	532 (34.6%)	299 (32.1%)	172 (36.8%)	61 (44.2%)	0.01*	0.084	0.005**	0.114
Gender					<0.0001***	<0.0001***	0.892	0.011*
Male, n(%)	739 (48.1%)	480 (51.6%)	187 (40%)	72 (52.2%)				
Female, n(%)	798 (51.9%)	451 (48.4%)	281 (60%)	66 (47.8%)				
HB, g/L media (range)	175 (38‐261)	194 (156‐261)	143 (39‐159)	109 (38‐158)	<0.0001***	<0.0001***	<0.0001***	<0.0001***
HCT media (range)	52.5 (10.4‐80.4)	59.4 (46‐80.4)	43.55 (20.5‐47.2)	34.6 (10.4‐46.5)	<0.0001***	<0.0001***	<0.0001***	<0.0001***
WBC, ×10^9^/L media (range)	11.6 (1.05‐68.5)	12.31 (1.05‐68.5)	10.20 (3.36‐53.4)	11.13 (1.29‐63.9)	<0.0001***	<0.0001***	0.06	0.513
PLT, ×10^9^/L media (range)	522 (9‐3772)	420 (100‐2138)	779 (550‐3760)	239.5 (9‐3772)	<0.0001***	<0.0001***	<0.0001***	<0.0001***
Abnormal karyotype(n = 1238)	90 (7.3%)	56 (698)(8.02%)	18 (423)(4.3%)	16 (117)(13.7%)	0.001**	0.021*	0.034*	<0.0001***
*V617F%*≥50%(n = 270)	87 (32.2%)	68 (183)(37.2%)	7 (66)(10.6%)	12 (21)(57.1%)	<0.0001***	<0.0001***	0.152	<0.0001***
Palpable splenomegaly, n(%)	925 (60.2%)	644 (69.2%)	167 (35.7%)	114 (82.6%)	<0.0001***	<0.0001***	0.001**	<0.0001***
Hypertension n(%)	593 (38.6%)	410 (44%)	153 (32.7%)	30 (21.7%)	<0.0001***	<0.0001***	<0.0001***	0.014*
Hyperlipidemia n(%)	216 (14.1%)	150 (16.1%)	56 (12%)	10 (16%)	0.006**	0.039*	0.006**	0.118
Diabetes n(%)	159 (10.3%)	94 (10.1%)	45 (9.6%)	20 (14.5%)	0.236	0.777	0.119	0.104
Smoking n(%)	150 (9.8%)	99 (10.6%)	38 (8.1%)	13 (9.4%)	0.324	0.136	0.664	0.629
At least one CV n(%)	796 (51.8%)	532 (57.1%)	209 (44.7%)	55 (39.%)	<0.0001***			
Thrombosis n(%)	675/1537 (43.9%)	439/931 (47.2%)	197/468 (42.1%)	39/138 (28.3%)	<0.0001***	0.073	<0.0001***	0.003**
Arterial thrombosis n(%)	617/675 (91.4%)	410/439 (93.4%)	172/197 (87.4%)	35/39 (89.8%)	<0.0001***	0.009**	<0.0001 ***	0.013*
ACS n(%)	156/617 (25.3%)	97/410 (23.7%)	52/172 (30.2%)	7/35 (20%)	0.092	0.692	0.048*	0.036*
TIA n(%)	454/617 (73.6%)	310/410 (75.7%)	121/172 (70.3%)	23/35 (65.7%)	<0.0001***	0.088	<0.0001***	0.026*
Splenic thrombosis n(%)	40/617 (6.5%)	28/410 (6.8%)	6/172 (3.5%)	6/35 (17.1%)	0.047*	0.004**	0.403	0.023*
Peripheral arterial n(%)	26/617 (4.2%)	19/410 (4.6%)	6/172 (3.5%)	1/35 (2.9%)	0.529	0.312	0.287	0.591
Others n(%)	14/617 (2.3%)	7/410 (1.7%)	7/172 (4.1%)	0	0.247	0.187	0.307	0.149
Venous thrombosis n(%)	112/675 (16.6%)	67/439 (15.3%)	39/197 (19.8%)	6/39 (15.4%)	0.292	0.449	0.216	0.117
Peripheral venous n(%)	79/112 (70.5%)	49/67 (73.1%)	26/39 (66.7%)	4/6 (66.7%)	0.501	0.819	0.233	0.206
Budd‐Chiari syndrome n(%)	21/112 (18.8%)	12/67 (17.9%)	7/39 (17.9%)	2/6 (33.3%)	0.84	0.753	0.877	0.968
PE n(%)	7/112 (6.2%)	5/67 (7.5%)	2/39 (5.1%)	0	1	0.784	0.388	0.442
Cerebral sinus thrombosis n(%)	3/112 (2.7%)	0	3/39 (7.7%)	0	0.076	0.014**	1	0.346
Others n(%)	5/112 (4.5%)	3/67 (4.5%)	2/39 (5.1%)	0	1	0.756	0.504	0.442
Thrombosis before diagnosis n(%)	431/675 (63.9%)	276/439 (62.9%)	136/197 (69%)	19/39 (48.7%)	<0.0001***	0.821	<0.0001***	<0.0001***
Thrombosis after diagnosis n(%)	354/675 (52.4%)	235/439 (53.5%)	94/197 (47.7%)	25/39 (64.1%)	0.035*	0.032*	0.069	0.609
Number of thrombosis n(%)					0.274	0.358	0.104	0.248
1 time n(%)	452 (66.9%)	285/439 (64.9%)	136/197 (69%)	31/39 (79.5%)				
2 times n(%)	153 (22.7%)	108/439 (24.6%)	41/197 (20.8%)	4/39 (10.3%)				
≥3 times n(%)	70 (10.4%)	46/439 (10.5%)	20/197 (10.2%)	4/39 (10.3%)				
Progression to AML n(%)	38 (2.5%)	20 (2.1%)	3 (0.6%)	15 (10.9%)	<0.0001***	0.037 *	<0.0001***	<0.0001***
Progression to MF(n = 1399)	259 (16.8%)	195 (20.9%)	64 (13.7%)		0.001**	0.001**		
Death n(%)	197 (12.8%)	115 (13.4%)	26 (5.6%)	56 (40.6%)	<0.0001***	<0.0001***	<0.0001***	<0.0001***
ASP therapy	914 (59.5%)	551 (59.2%)	335 (71.6%)	28 (20.3%)	<0.0001***	<0.0001***	<0.0001***	<0.0001***

Abbreviations:; ACS, acute coronary syndrome; AML, acute myeloid leukemia; ASP, aspirin; CV, risk factors for cardiovascular events; ET, essential thrombocythemia; HB, hemoglobin; HCT, hematocrit; *JAK2^V617F^* allele burden; MF, myelofibrosis; PE, pulmonary embolism, PLT, platelet count; PMF, primary myelofibrosis; PV, polycythemia vera; TIA, transient ischemic attack; WBC, white blood cell; *V617F%*.

* *P* < .05.

** *P* < .01.

*** *P* < .0001.

Thrombotic events occurred in 43.9% (675/1537) of patients. Among 1399 patients with PV and ET, 259 (16.8%) had secondary bone marrow fibrosis. The incidence of secondary myelofibrosis in PV patients (20.9%) was significantly higher than that in ET patients (13.7%) (*P* = .001). A total of 2.5% of patients (38) developed acute myeloid leukemia (AML), and the AML transformation rate in PMF patients (10.9%) was significantly higher than that in PV (2.1%) and ET (0.6%) patients (*P* < .0001) (Table 1). A total of 197 patients (12.8%) died, among whom 23.4% died of thrombosis and 19.3% died of AML transformation (Table S1).

### 3.2 Survival of *JAK2^V617F^*‐mutated MPN patient

**Figure 1:**
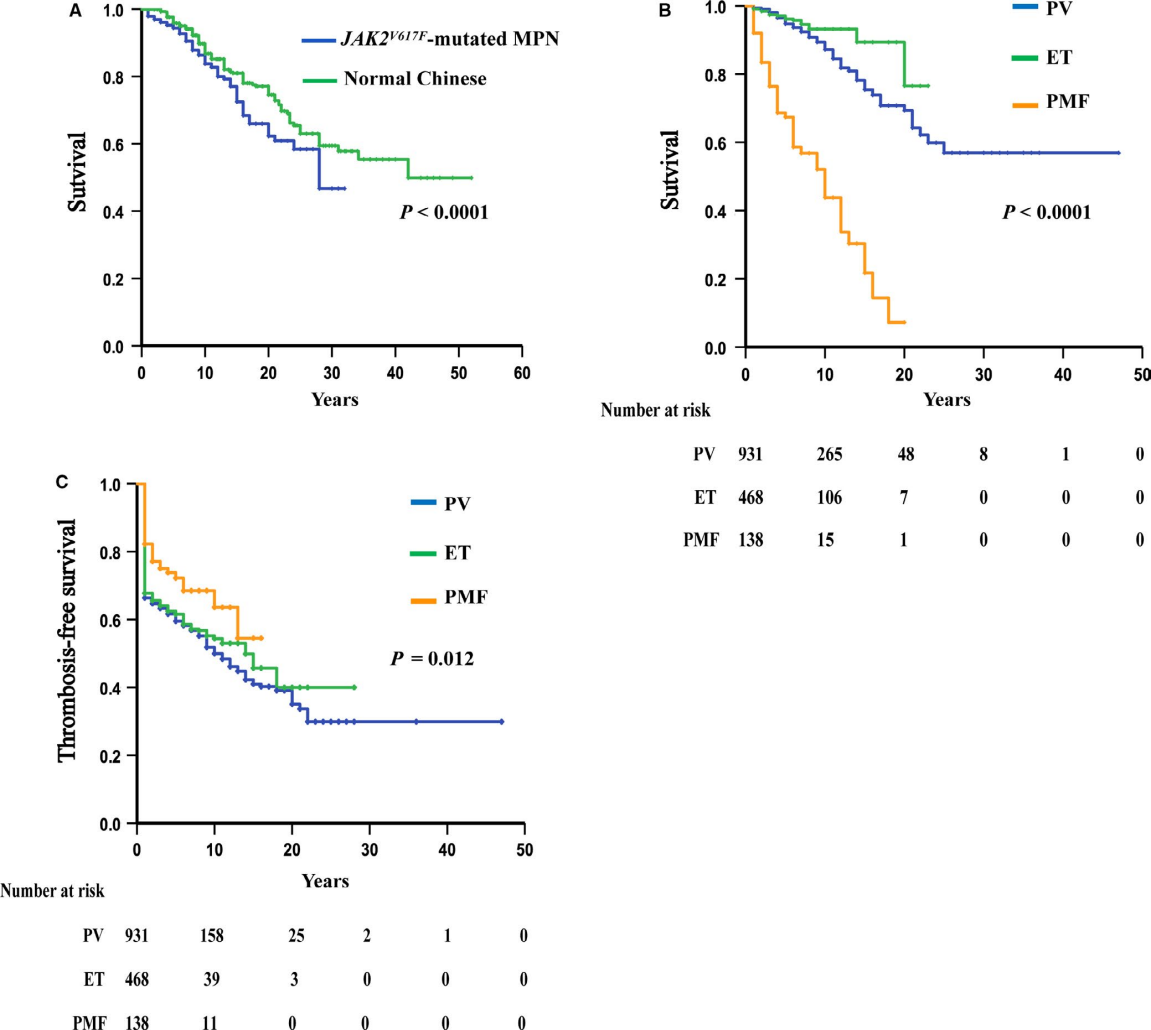
Overall survival of patients with *JAK2^V617F^*‐mutated MPN. A, Comparison of overall survival of patients with *JAK2^V617F^*‐mutated MPN with healthy Chinese individuals. B, Comparison of the overall survival of patients with *JAK2^V617F^*‐mutated PV, ET and PMF. C, Comparison of thrombosis‐free survival of patients with *JAK2^V617F^*‐mutated PV, ET and PMF

The median follow‐up time for the 1537 patients with *JAK2^V617F^*‐mutated MPN was 7 years (1 to 47 years). The median survival time of patients with *JAK2^V617F^‐*mutated MPN was 27 years, and the 10‐year, 15‐year, and 20‐year survival rates were 85.5%, 73.6%, and 66.2%, respectively. The patient mortality rate was 1.7/100 (95% CI [1.67/100, 2.01/100]) patient‐years. The OS of patients with *JAK2^V617F^*‐mutated MPN was significantly worse than that of healthy Chinese individuals matched by age and sex, with an SMR of 2.24 (95% CI [1.28, 3.20], *P* < .0001) (Figure 1A). For patients with *JAK2^V617F^‐*mutated MPN with different diagnoses, the OS of PMF patients was significantly worse than that of PV and ET patients; ET patients had the best OS (Figure 1B).

### 3.3 Characteristics of thrombosis in patients with *JAK2^V617F^*‐mutated MPN

A total of 43.9% (675/1537) of MPN patients had a thrombotic event, corresponding to an incidence of 5.82/100 (95% CI [5.72/100, 6.89/100]) person/year. *JAK2^V617F^‐*mutated MPN patients with thrombosis were older; had higher hematocrit (HCT), hemoglobin, and white blood cell counts; had a higher incidence of cardiovascular (CV) risk factors, had a higher *V617F*% (*P* < .05). Approximately 70.9% patients with *V617F% *≥50% developed thrombosis, which was significantly higher than the incidence of patients with *V617F% *≥50% who did not develop thrombosis (*P* < .0001). The incidence of thrombosis was different in patients with different grades of bone marrow fibrosis (*P* = .002) and was higher in patients with bone marrow fibrosis grade 1 or 2 than in patients with higher grade fibrosis (Table 2).

**Table 2:** Comparison of clinical characteristics of patients with *JAK2^V617F^*‐mutated MPN with or without thrombosis

	NO thrombosis(n = 862)	Thrombotic events(n = 675)	*P*
Age,media (range)	52 (18‐89)	58 (22‐86)	<0.0001***
Age ≥ 60 (n = 532)%	240 (45.1%)	292 (54.9%)	<0.0001***
Male, n (%)	386 (44.8%)	353 (52.3%)	0.004**
HB,g/L media (range)	170 (39‐253)	182 (38‐261)	0.001**
HCT ≥ 45% (n = 1065)%	575 (53.9%)	490 (46.1%)	0.014*
HCT ≥ 48% (n = 675)%	226 (33.5%)	449 (66.5%)	0.002**
WBC × 10^9^/L media (range)	11.33 (1.39‐63.9)	12 (1.05‐68.5)	0.004**
PLT × 10^9^/L media (range)	510 (9‐3760)	532 (10‐3772)	0.171
Abnormal karyotype (n = 1238)%	44 (n = 717)(6.1%)	46 (n = 521)(8.8%)	0.077
*V617F%*≥50%(n = 87)%	26 (29.9%)	61 (70.9%)	<0.0001***
Reticulin = 0 (n = 788)%	465 (59.1%)	323 (40.9%)	0.002**
Reticulin = 1 (n = 365)%	180 (49.3%)	185 (50.7%)	
Reticulin = 2 (n = 146)%	72 (49.3%)	74 (50.7%)	
Reticulin = 3 (n = 238)%	145 (60.9%)	93 (39.1%)	
Diagnosis			<0.0001***
PV (n = 931) %	492 (52.8%)	439 (47.2%)	
ET(n = 468) %	271 (57.9%)	197 (42.1%)	
PMF (n = 138) %	99 (71.7%)	39 (28.3%)	
Palpable splenomegaly (n = 925) %	523 (56.5%)	402 (43.5%)	0.675
Hypertension(n = 593) %	247 (41.7%)	346 (58.3%)	<0.0001***
Hyperlipidemia(n = 216) %	82 (37.9%)	134 (62.1%)	<0.0001***
Diabetes(n = 159) %	65 (40.9%)	94 (59.1%)	<0.0001***
Smoking(n = 150) %	68 (45.3%)	82 (54.7%)	0.006**
At least one CV (n = 796)%	354 (44.5%)	442 (55.5%)	<0.0001***
Bleeding(n = 79) %	36 (45.6%)	43 (54.4%)	0.062
Death(n = 197) %	79 (40.1%)	118 (59.9%)	<0.0001***

* *P* < .05.

** *P* < .01.

*** *P* < .0001.

The incidence of arterial thrombosis (91.4%, 617 patients) was significantly higher than that of venous thrombosis (16.6%, 112 patients). Cerebrovascular accidents (73.6%) were the main cause of arterial thrombosis. The incidence of thrombosis at the time of diagnosis or before diagnosis was 63.9% (431/675), and 52.4% (354/675) of patients still developed thrombosis after diagnosis. A total of 33.1% (223/675) of patients experienced two or more thrombotic events (Table 1).

The overall incidence of thrombosis in patients with PV and ET was higher than that in patients with PMF (*P* < .0001, *P* = .003), and the incidence of thrombosis was higher in PV (62.9%) and ET (69%) patients at or before diagnosis; however, more thrombosis occurred in PMF (64.1%) patients after diagnosis. Furthermore, PMF had the highest incidence of splenic infarction (17.1%) (Table 1).

To establish a prognostic model, we selected the cutoff value determined by the ROC curve and applied it to conduct a univariate analysis for continuous variables such as HCT, white blood cell count, and platelet count. The indicators with statistical significance in the univariate analysis (age ≥60 years, male, HCT ≥48%, white blood cell count ≥10 × 10^9^/L, platelet count ≥ 475 × 10^9^/L, at least one CV risk factor, reticular fibers, history of thrombosis, and *V617F% *≥50%) were included in the multivariable analysis. The multivariable analysis indicated that age ≥60 years old (*P* = .003, HR = 1.76, 95% CI [1.214, 2.552]), HCT ≥48% (*P* = .022, HR = 1.635, 95% CI [1.073, 2.492]), at least one CV risk factor (*P* = .024, HR = 1.559, 95% CI [1.061,2.291]), a history of thrombosis (*P* < .0001, HR = 2.313, 95% CI [1.573,3.401]), and *JAK2^V617F^* allele burden (*V617F*%) ≥50% (*P* = .003, HR = 1.804, 95% CI [1.221, 2.665]) are risk factors for thrombosis of *JAK2^V617F^*‐mutated MPN (Table 3, Table S2).

**Table 3:** Risk model of thrombosis in patients with *JAK2^V617F^*‐mutated MPN

	Multivariate analysis			
	HR	95%CI	*P*	
Age ≥60	1.76	1.214,2.552	0.003**	1 point
At least one CV	1.559	1.061,2.291	0.024*	1 point
HCT ≥48%	1.635	1.073,2.492	0.022*	1 point
History of thrombosis	2.313	1.573,3.401	<0.0001***	1 points
*V617F% *≥50%	1.804	1.221,2.665	0.003**	1 points

Note

low‐risk (0 points), intermediate‐risk (1 points) and high‐risk(≥2 points).

According to a detailed analysis of the site of thrombosis, V617F% ≥50% (*P* = .01, HR = 1.705, 95% CI [1.134,2.564], age ≥60 years old (*P* < .0001,HR = 2.07, 95% CI [1.406,3.047]), male (*P* = .045, HR = 1.456, 95% CI [1.008, 2.102]), HCT ≥ 48% (*P* = .009, HR = 1.742, 95% CI [1.148,2.643]), at least one CV risk factor (*P* = .036, HR = 1.544, 95% CI [1.028, 2.317]) and a history of thrombosis (*P* = .582, 95% CI [1.682]) were risk factors affecting arterial thrombosis in MPN patients. However, only a history of thrombosis (*P* < .0001, HR = 8.339, 95% CI [3.656, 19.021]) was a risk factor affecting venous thrombosis in MPN patients (Table S3).

**Figure 2:**
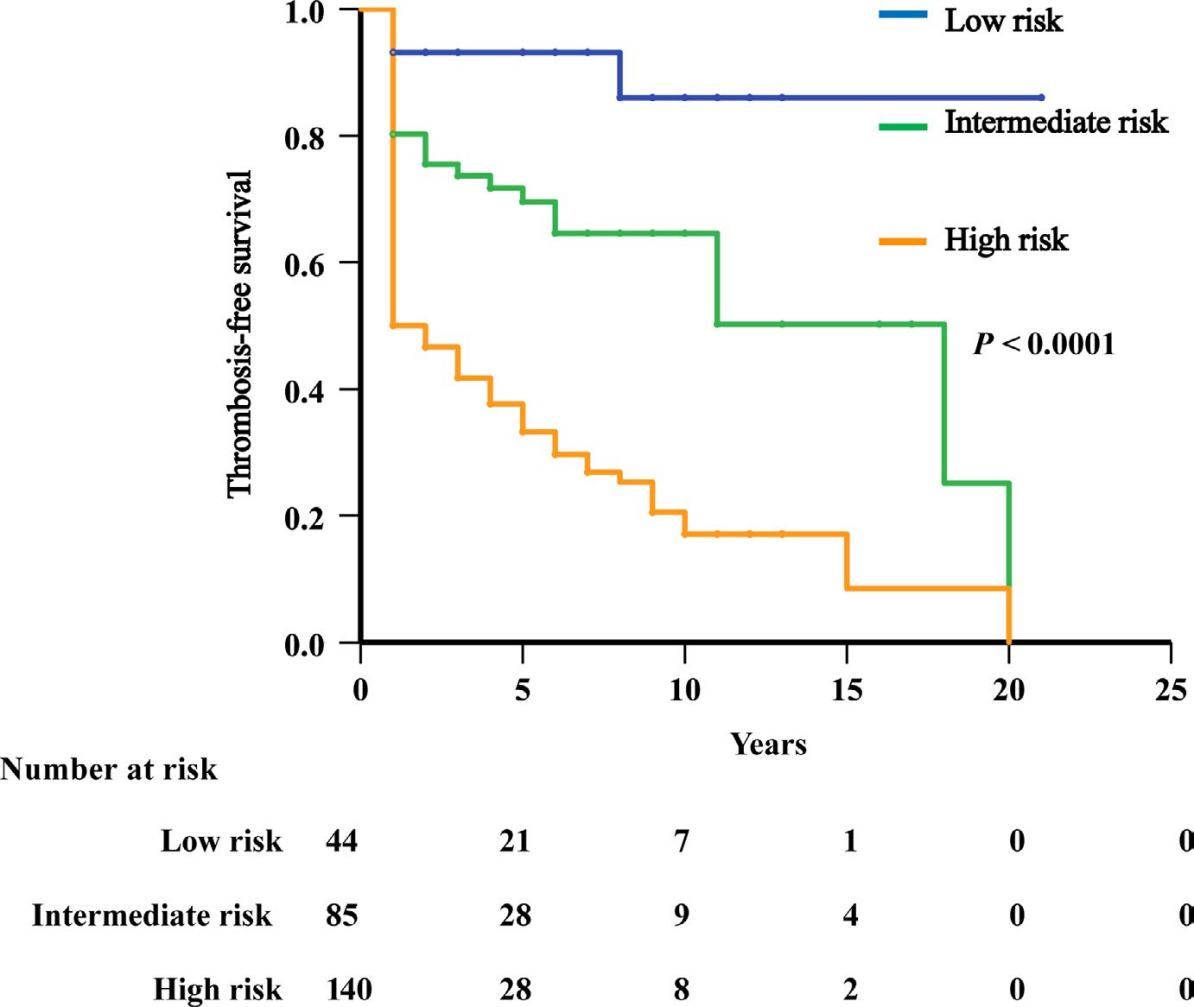
Thrombosis‐free survival of patients with *JAK2^V617F^*‐mutated MPN stratified by a new risk model of thrombosis. Patients were stratified into low‐risk (0 points), intermediate‐risk (1 points) and high‐risk (≥2 points) groups

Based on the results of the multivariable analysis, a predictive model was devised with the following risk factors: age ≥60 years old, at least one CV risk factor, history of thrombosis, and *V617F*% ≥50%. Weighted adverse scores were obtained according to the HR values of the different risk factors: age ≥60 years old (1 point), HCT ≥48% (1 point), at least one CV risk factor (1 point), history of thrombosis (1 points), and *V617F*% ≥50% (1 points), and the risk model was established. The groups included a low‐risk (0 points), intermediate‐risk (1 points), and high‐risk group (≥2 points). Using this new risk model of thrombosis, we observed that the incidence of thrombosis was 72.9%, 33.7%, and 9.1% in the high‐, intermediate‐ and low‐risk groups, respectively, and was significantly different between each group (*P* < .0001). The results of thrombosis‐free survival analysis showed that patients in the high‐risk group (n = 140; average thrombosis‐free survival, 5 years; 95% CI [4.16,6.64]), intermediate‐risk group (n = 86; average thrombosis‐free survival, 11 years; 95% CI [9.578,14.333]), and low‐risk group (n = 44; average thrombosis‐free survival, 18 years; 95% CI [16.45,20.959]) had vastly different thrombosis‐free survival times (Figure 2).

## 4 DISCUSSION

It has been reported that the incidence of thrombosis in PV, ET, and PMF patients was 5.5, 3.0, and 2.23 per 100 patient‐years.^14^, ^15^, ^16^ Thrombosis‐related deaths accounted for 45%, 26%, and 12% of deaths in patients with PV, ET, and PMF^17^, ^18^, ^19^; therefore, thrombosis is considered the most important cause of death and disability of MPN patients, and the study of thrombosis is a consistent topic in the field of MPN.

Multiple studies have shown a clear correlation between different driver mutations (*JAK2*/*CALR*/*MPL*) and thrombosis in MPN patients.^20^, ^21^, ^22^ For example, PMF patients with *JAK2^V617F^* and *MPL^W515L/K^* mutations have a higher risk of thrombosis and worse prognosis than patients with *CALR* mutations, and *CALR* type I mutations corresponded to better prognosis than *CALR* type II mutations.^9^, ^22^ Previous studies have focused on the occurrence of thrombosis in the context of the same disease subtype (PV or ET or PMF), but this study focused on the same gene mutation (*JAK2^V617F^*) among different subtypes of MPN to study the thrombosis of different diagnoses of MPN, which can exclude the influence of other driver mutations. By studying this particular driver mutation, *JAK2^V617F^*, we can explore the characteristics in MPN patients who have thrombosis and varying clinical characteristics, identify common risk factors for thrombosis in patients with *JAK2^V617F^*‐mutated MPN, and provide a theoretical basis for accurately preventing and treating thrombosis in patients with *JAK2^V617F^*‐mutated MPN. Our study found that the overall incidence of thrombosis in patients with *JAK2^V617F^*‐mutated MPN was 5.82/100 patient‐years, which was higher than that in patients with MPN with other driver gene mutations, confirming the importance of the *JAK2^V617F^* mutation in the formation of thrombosis.

Regarding the site of thrombosis, Hultcrantz^23^ reported that the incidence of arterial thrombosis in PV and ET patients was approximately 2‐3 times higher than that of venous thrombosis, but the incidence of arterial and venous thrombosis in PMF patients was similar. In our study, the incidence of arterial thrombosis (91.4%) in patients with *JAK2^V617F^*‐mutated MPN was higher than that observed in Western‐based studies.^24^ The incidence of venous thrombosis (16.6%) is lower, and it is possible that ethnically specific inflammatory cytokines affect coagulation and are related to the different observations by physicians with regard to the latent incidence of venous thrombosis.

We further refined the analysis of thrombosis in patients with *JAK2^V617F^*‐mutated MPN with different subtypes, and the results show that the risk of thrombosis in PV patients was significantly higher than that in ET and PMF patients. For PV patients, similar to the study by Tefferi A et al,^25^ age ≥60 years, history of thrombosis and *V617F% *≥50% were risk factors affecting PV thrombosis, especially arterial thrombosis. In PV patients, the incidence of thrombosis in patients with *V617F% *≥50% was 4.632 times higher than that in patients with *V617F% *<50%. Therefore, reducing *V617F%* during the treatment of PV patients is crucial to prevent thrombosis, especially the occurrence of arterial thrombosis complications. Compared with PV and ET patients, PMF patients had a relatively lower overall incidence of thrombosis, which may be related to the higher progression rate and early mortality of acute leukemia transformation and other diseases in PMF patients. In this study, the high incidence of portal vein and splenic arterial thrombosis in PMF was associated with significant splenomegaly and more complex inflammatory factors, which trigger abnormal coagulation.

HCT and reticular fiber quantity are important indicators for the classification and diagnosis of MPN. Tiziano and Roberto^26^ showed that PV patients with 45%‐50% HCT had a significantly higher risk of thrombosis than those with controlled HCT at 45%. In our study, the HCT cutoff value that affected thrombosis was 48%. We then found that only 46.1% of patients with HCT ≥45% had thrombosis, whereas 66.5% of patients with HCT ≥48% had thrombosis. Multivariate analysis showed that HCT ≥48% was a risk factor for thrombosis in *JAK2^V617F^*‐mutated MPN. Campbell et al^27^ showed that with an increase in the number of reticular fibers, the incidence of thrombosis in MPN also increases. In our study, it was found that for patients with *JAK2^V617F^*‐mutated MPN, although the incidence of thrombosis increased with the increase in the number of grade 0‐2 reticular fibers, the incidence of thrombosis was lower in patients with grade 3 reticular fibers. This may be associated with a higher incidence of acute leukemia transformation and early mortality in patients with a higher number of reticular fibers.

CV risk factors mainly include hypertension, hyperlipidemia, diabetes, and smoking. Our results showed that 55.5% of patients with thrombosis had at least one CV risk factor, and at least one CV risk factors was also a risk factor for thrombosis in Chinese PV patients. Multivariate analysis also demonstrated the importance of CV risk factors. Thus, CV risk factors play an important role in the prognosis of thrombosis in patients with *JAK2^V617F^*‐mutated MPN.

This study shows that a history of thrombosis is a risk factor for thrombosis in *JAK2^V617F^*‐mutated MPN, whether in PV, ET or PMF. Therefore, in the treatment of patients with *JAK2^V617F^*‐mutated MPN, more active measures, such as cytoreductive and anti‐atherosclerosis regimens, should be taken for those patients who have experienced thrombosis to prevent the recurrence of thrombosis and improve survival.

Tefferi A^14^ and Barbui T^12^ established the thrombosis prognostic models of PV and ET, respectively, and their results showed that a history of thrombosis is an important risk factor for MPN patients. On the basis of our multivariable analysis, a risk model of Chinese MPN patients with the *JAK2^V617F^* mutation was established based on age ≥60 years old, HCT ≥48%, at least one cardiovascular risk factor, history of thrombosis, and *V617F*% ≥50%. Furthermore, this model showed that the incidence of thrombosis in the high‐risk group was as high as 72.9%, and average thrombosis‐free survival was 5 years. Therefore, for high‐risk patients, reducing *V617F%*, controlling HCT and mitigating cardiovascular risk factors are necessary measures to prevent thrombosis in patients with *JAK2^V617F^*‐mutated MPN.

In conclusion, the Chinese thrombosis risk model of *JAK2^V617F^‐*mutated MPN established in this study suggests that elderly patients with a history of thrombosis should reduce *V617F%*, control HCT to less than 48% and mitigate cardiovascular risk factors, all of which are necessary measures to prevent thrombosis in patients with *JAK2^V617F^*‐mutated MPN. As the application of deep‐sequencing technology has advanced the diagnosis and prognosis evaluation of hematologic diseases, our future research direction is to explore the role of different accompanying gene mutations in thrombotic events of patients with *JAK2^V617F^*‐mutated MPN and to establish a thrombosis model that includes accompanying mutations.

## CONFLICTS OF INTEREST

The authors declare no conflicts of interests.

## Data Availability

Our data is available.
