# Supplementary material for: Thrombosis among 1537 patients with JAK2V617F‐mutated myeloproliferative neoplasms: Risk factors and development of a predictive model
Source: Cancer Med. 2020 Jan 28;9(6):2096–105. doi: 10.1002/cam4.2886 (PMC7064115; doi:10.1002/cam4.2886)
Supplement: Supplementary file 1 [file CAM4-9-2096-s001.docx]

**Supplementary Table S1:** **Cause of death in patients with *JAK2^V617F^*-mutated MPN.**

| **Cause of death** | **Total（n=1537）** | **PV(n=931)** | **ET(n=468)** | **PMF(n=138)** | **P** | **PV&ET** | **PV&PMF** | **ET&PMF** |
| --- | --- | --- | --- | --- | --- | --- | --- | --- |
| **AML n(%)** | **38/197(19.3%)** | **20/115(17.4%)** | **3/26(11.5%)** | **15/56（26.8%）** | **0.193** | **0.467** | **0.154** | **0.123** |
| **Organ failure n(%)** | **33/197(16.8%)** | **13/115(11.3%)** | **7/26(26.9%)** | **13/56（23.2%）** | **0.048** | **0.04** | **0.042** | **0.718** |
| **Thrombosis n(%)** | **46/197(23.4%)** | **36/115(31.3%)** | **3/26(11.5%)** | **7/56（12.5%）** | **0.008** | **0.043** | **0.008** | **0.902** |
| **Cancer n(%)** | **27/197(13.7%)** | **14/115(12.2%)** | **4/26(15.4%)** | **9/56（16.1%）** | **0.758** | **0659** | **0.485** | **0.937** |
| **Infection n(%)** | **7/197(3.6%)** | **5/115(4.3%)** | **0** | **2/56（3.6%）** | **0.557** | **0.281** | **0.811** | **0.322** |
| **Beelding n(%)** | **14/197(7.1%)** | **7/115(6.1%)** | **3/26(11.5%)** | **4/56（7.1%）** | **0.567** | **0.330** | **0.792** | **0.510** |
| **Unknown n(%)** | **32/197(16.2%)** | **20/115(17.4%)** | **6/26(23.1%)** | **6/56（10.7%）** | **0.315** | **0.501** | **0.255** | **0.143** |

PV, polycythemia vera; ET, essential thrombocythemia; PMF, primary myelofibrosis; AML, acute myeloid leukemia.

**Supplementary Table S2:** **Risk factors of thrombosis in patients with *JAK2^V617F^*-mutated MPN.**

|  | **Univariate analysis** | | **Multivariate analysis** | | |
| --- | --- | --- | --- | --- | --- |
|  | **Chi-Square value** | **P** | **HR** | **95%CI** | **P** |
| **Age≥60** | **69.303** | **<0.0001** | **1.76** | **1.214，2.552** | **0.003** |
| **Male** | **10.295** | **0.001** | **1.399** | **0.988，1.98** | **0.058** |
| **HCT≥48%** | **4.179** | **0.041** | **1.635** | **1.073,2.492** | **0.022** |
| **WBC≥10×10^9^/L** | **4.029** | **0.045** | **0.982** | **0.676，1.425** | **0.922** |
| **PLT≥475×10^9^/L** | **4.888** | **0.027** | **1.163** | **0.797，1.698** | **0.433** |
| **At least one CV** | **74.227** | **<0.0001** | **1.559** | **1.061，2.291** | **0.024** |
| **History of thrombosis** | **417.011** | **<0.0001** | **2.313** | **1.573，3.401** | **<0.0001** |
| **Reticulin** | **16.11** | **0.001** | **0.904** | **0.757，1.079** | **0.263** |
| ***V617F%*≥50%** | **13.997** | **<0.0001** | **1.804** | **1.221，2.665** | **0.003** |

HCT, hematocrit; PLT, platelet count; WBC, white blood cell; CV, risk factors for cardiovascular events; *V617F%*, *JAK2^V617F^* allele burden.

**Supplementary Table S3: Risk factors of arterial or venous thrombosis in patients with *JAK2^V617F^*-mutated MPN.**

| **Arterial thrombosis** | **Univariate analysis** | | **Multivariate analysis** | | |
| --- | --- | --- | --- | --- | --- |
|  | **Chi-Square value** | **P** | **HR** | **95%CI** | **P** |
| **Age≥60** | **72.128** | **<0.0001** | **2.07** | **1.406,3.047** | **<0.0001** |
| **Male** | **13.399** | **<0.0001** | **1.456** | **1.008,2.102** | **0.045** |
| **HCT≥48%** | **7.879** | **0.005** | **1.742** | **1.148,2.643** | **0.009** |
| **WBC≥10×10^9^/L** | **7.533** | **0.006** | **1.172** | **0.795,1.728** | **0.422** |
| **PLT≥475×10^9^/L** | **2.876** | **0.09** |  |  |  |
| **At least one CV** | **71.123** | **<0.0001** | **1.544** | **1.028,2.317** | **0.036** |
| **History of thrombosis** | **436.868** | **<0.0001** | **2.514** | **1.682,3.758** | **<0.0001** |
| **Reticulin** | **15.388** | **0.002** | **0.912** | **0.757,1.098** | **0.33** |
| ***V617F%*≥50%** | **12.745** | **<0.0001** | **1.705** | **1.134,2.564** | **0.01** |
|  | **Univariate analysis** | | **Multivariate analysis** | | |
| **Venous thrombosis** | **Chi-Square value** | **P** | **HR** | **95%CI** | **P** |
| **Age≥60** | **1.876** | **0.171** |  |  |  |
| **Male** | **0.002** | **0.962** |  |  |  |
| **HCT≥48%** | **0.510** | **0.475** |  |  |  |
| **WBC≥10×10^9^/L** | **0.698** | **0.403** |  |  |  |
| **PLT≥475×10^9^/L** | **0.005** | **0.823** |  |  |  |
| **At least one CV** | **13.715** | **<0.0001** | **0.798** | **0.359,1.774** | **0.58** |
| **History of thrombosis** | **232.743** | **<0.0001** | **8.339** | **3.656,19.021** | **<0.0001** |
| **Reticulin** | **2.358** | **0.502** |  |  |  |
| ***V617F%*≥50%** | **5.051** | **0.025** | **1.349** | **0.617,2.949** | **0.453** |
